# Pharmacological Action of a Pregnane Glycoside, Russelioside B, in Dietary Obese Rats: Impact on Weight Gain and Energy Expenditure

**DOI:** 10.3389/fphar.2018.00990

**Published:** 2018-08-30

**Authors:** Essam Abdel-Sattar, Eman T. Mehanna, Sabah H. El-Ghaiesh, Hala M. F. Mohammad, Hanan A. Elgendy, Sawsan A. Zaitone

**Affiliations:** ^1^Department of Pharmacognosy, Faculty of Pharmacy, Cairo University, Cairo, Egypt; ^2^Department of Biochemistry, Faculty of Pharmacy, Suez Canal University, Ismailia, Egypt; ^3^Department of Pharmacology, Faculty of Medicine, Tanta University, Tanta, Egypt; ^4^Department of Clinical Pharmacology, Faculty of Medicine, Suez Canal University, Ismailia, Egypt; ^5^Department of Anatomy and Embryology, Faculty of Medicine, Mansoura University, Mansoura, Egypt; ^6^Department of Anatomy, Faculty of Medicine, Northern Border University, Arar, Saudi Arabia; ^7^Department of Pharmacology and Toxicology, Faculty of Pharmacy, Suez Canal University, Ismailia, Egypt; ^8^Department of Pharmacology and Toxicology, Faculty of Pharmacy, University of Tabuk, Tabuk, Saudi Arabia

**Keywords:** russelioside B, *Caralluma quadrangula*, diet-induced obesity, energy expenditure, high fat diet, insulin resistance, adipokines, pro-inflammatory cytokines

## Abstract

**Background and purpose:** Russelioside B (RB) is a pregnane glycoside obtained from *Caralluma quadrangula*; a herb with antidiabetic, anti-inflammatory, and antihyperlipidemic activities. The present experiment tested the possible role of RB in controlling weight gain in rats fed on high fat (HF) diet.

**Methods:** RB was separated from the n-butanol fraction of the crude methanolic extract by chromatographic separation on a Si gel column according to the procedures described previously. The experiment of the biological assessment of RB used 32 male Wistar rats (4 groups, *n* = 8). Group 1 rats were fed with a palatable normal diet. Group 2, 3, and 4 were fed on HF diet for 16 weeks. Group 2 served as the HF diet control group while Group 3 and 4 received daily oral doses of RB (25 and 50 mg/kg) during the last four weeks. Animals’ parameters like weight gain, fasting level of blood sugar, serum lipids, and serum liver enzyme activities were measured. Liver or adipose tissue weight was divided by the rat’s body weight and multiplied by 100 to obtain the liver or adipose tissue index, respectively. Adipose tissues were processed for histopathological examination, measurement of mRNA expression of visfatin, leptin, adiponectin, uncoupling protein-1 (UCP-1), and carnitine palmitoyl transferase-1 (CPT-1). Furthermore, serum levels of insulin, interleukin-6 (IL-6), IL-1β, tumor necrosis factor-α (TNF-α), leptin, resistin, and adiponectin were assessed using ELISA kits.

**Results:** Rats fed with the HF diet exhibited significant body weight gain, abnormal liver function, disturbed lipid profile, and greater serum level of pro-inflammatory cytokines in addition to greater insulin resistance, adipose tissue and liver indices. Further, rats fed with the HF diet displayed upregulations in the expression of visfatin and leptin with downregulations in the expression of adiponectin, UCP-1, and CPT-1 compared to normal rats. Interestingly, RB (25 or 50 mg/kg) favorably modulated the measured parameters.

**Conclusion:** Data from this study documented the beneficial role of RB in diminishing weight gain, improving the inflammatory perturbations and energy expenditure in HF diet fed rats. Therefore, RB might be a promising candidate for obesity.

## Introduction

Diet-induced obesity (DIO) and insulin resistance are clinical executions of metaflammatory derangements foreboding type-2 diabetes mellitus (Wellen and Hotamisligil, 2005). The interplay of pro-inflammatory and metabolic disturbances is pivotal in for developing insulin resistance and its ensuing morbidities (Bilir et al., 2016). DIO is associated with increased inflammatory markers (Hosseinzadeh-Attar et al., 2016) and perturbations of adipose secretory hormones (adiponectin, visfatin, resistin, and leptin) (Trayhurn and Beattie, 2001; Simons et al., 2007). Leptin is an adipocyte-secreted hormone that interacts with neuronal pathways in the brain resulting in suppressing food consumption and enhancing energy expenditure. However, many cases of obese people have greater serum leptin versus lean individuals, this takes place without sufficient appetite suppression; a phenomenon known as leptin resistance (Crujeiras et al., 2015).

Adaptive thermogenesis is a life-essential homeostatic process. Uncoupling protein-1 (UCP-1) is expressed mainly by the brown adipose tissues. UCP-1 is sited within the inner mitochondrial membrane where it increases energy dissipation via uncoupling of mitochondrial oxidation of substrates producing heat instead of ATP (Bargut et al., 2016). Carnitine palmitoyl transferase-1 (CPT-1) is sited at the outer membrane of the mitochondria and it is the rate-determining step in fatty acid β-oxidation. Its role is to shuttle long-chain fatty acids from the cytoplasm to the mitochondrial inner membrane for further catabolism and energy production (Zammit, 2008).

Finding new therapeutic candidates targeting obesity is a challenging goal. Herbal remedies represent a community-popular and a preferential choice for life-long therapies. The genus *Caralluma* is rich in pregnane glycosides with well-established ethnomedicinal applications (Adnan et al., 2014). *Caralluma fimbriata* extract showed appetite suppressant and weight reducing effects in animal models (Preuss, 2004; Kamalakkannan et al., 2010; Ambadasu et al., 2013) and in overweight individuals and, it has been FDA approved as a slimming nutraceutical (Kuriyan et al., 2007).

**FIGURE 1:**
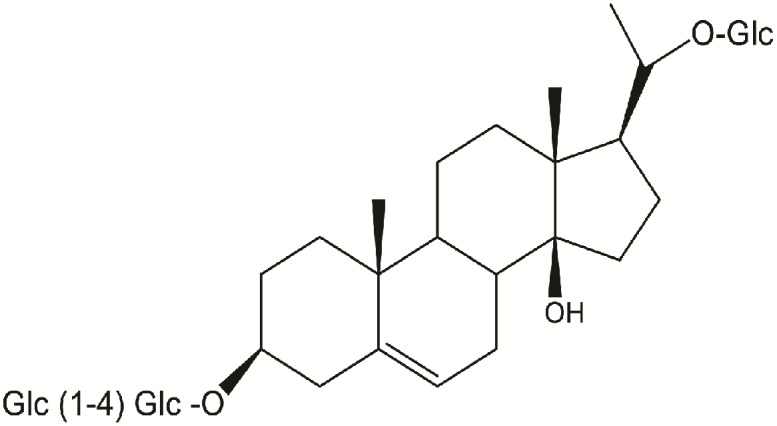
Chemical structure of russelioside B.

Russelioside B (RB), is a pregnane glycoside isolated from *C. russeliana* (Al-Yahya et al., 2000) and *C. quadrangula* (Abdel-Sattar et al., 2016; **Figure 1**). Further, non-published data from our laboratory indicated the presence of RB in the butanol fraction of *C. tuberculata*. In addition to RB, russeliosides A, C, and D and four acylated pregnane glycosides were isolated from *C. quadrangula* according to previous reports (Abdallah et al., 2013; Abdel-Sattar et al., 2016), all of them were isolated in a low yield (not more than 100 mg) which is not enough to perform an *in vivo* study, therefore RB was selected for the current study. In previous studies, RB or extracts rich in RB improved hyperglycemia in streptozotocin-diabetic rats (Abdel-Sattar et al., 2013, 2016).

The current study aimed to examine the potential role of RB in controlling weight gain associated with HF feeding in rats focusing on the influence of RB on adipose tissue expression for adipokines as well as energy expenditure.

## Materials and Methods

### Collection of the Plant Materials

Aerial parts of *C. quadrangula* (Forssk.) N. E. Br. (Syn. *Stapelia quadrangula* Forssk.) were collected from Al-Taif Governorate (Saudi Arabia), during April 2016 (end of fruiting stage) and were dried in shade. The plant material was authenticated by a staff member at the Taxonomy Department at the Faculty of Science in King Abdulaziz University. A specimen was deposited in the herbarium of College of Pharmacy, King Abdulaziz University, Jeddah, Saudi Arabia (# CQ 1027-B).

### Isolation of Russelioside B

In brief, the air-dried powdered aerial parts of *C. quadrangula* (480 g) were extracted with methanol (3 × 2 L) to give 82 g of brown residue. The methanolic extract (65 g) was partitioned into chloroform (8.8 g), and n-butanol (35.8 g). RB (calogenin 20-*O*-β-D-glucopyranosyl-3-*O*-[β-D-glucopyranosyl-(1→4)-β-D-(3-O-methyl-6-deoxy)galactoside]) was separated from the n-butanol fraction by chromatographic separation on a Si gel column according to the procedures described previously (Al-Yahya et al., 2000). RB purity was confirmed by superimposed IR and by comparison of its ^1^H-NMR (**Supplementary Figure S1**) and ^13^C-NMR (**Supplementary Figure S2**) with those reported in the literature (Al-Yahya et al., 2000).

### Biological Assays

#### The Composition of the High-Fat Diet

The HF diet consisted of standard feeding diet (87.7% wt/wt), pork fat (10% wt/wt), cholesterol (2% wt/wt), and bile salts (0.3% wt/wt) according to Pan et al. (2006). This diet is commonly used to induce insulin resistance and weight gain in rats.

#### Experimental Animals

Male Wistar rats (body weight = 110–170 g) were obtained from Moustafa Rashed Company for Laboratory Animals in Giza (Egypt). The experimental protocol of this study was agreed by the institutional research ethics committee at the Faculty of Pharmacy, Suez Canal University (Ismailia, Egypt) [license No. 201605RA1]. Experimental animals were kept in a clean well-ventilated animal house. Animals were maintained in polyethylene cages at 25 ± 5°C and normal dark/light cycle, with food and tap water provided *ad libitum*. Before starting experimentation, rats were left for an acclimatization period of 1–2 weeks.

#### Experimental Protocols

After adaptation, rats were assigned into four different groups (*n* = 8 rats) and fed on the following regimens for 16 weeks: (i) normal group: rats were fed on normal palatable diet; (ii) HF diet control group: rats were fed on the HF diet and received doses of the vehicle during the last four weeks (distilled water); (iii & iv) HF diet fed rats treated with RB (25 and 50 mg/kg) during the last four weeks of the experiment.

Russelioside B doses are similar to those used in a previous publication (Abdel-Sattar et al., 2016). In general, RB was administered *per os* (P.O.) in a volume equals 2 mL/kg in distilled water. Rats in group (i) received equal volumes of distilled water at the same time of the day. RB treatment was launched at the start of the 13^th^ week and continued until the last experimental day (the end of the 16^th^ week).

At end of 16^th^ week, rats were weighed and left fasting for an overnight for measurement of fasting blood glucose (FBG) through a tail vein puncture employing an automatic glucometer (Super Glucocard, Japan). Further, rats were anesthetized with thiopental sodium (50 mg/kg) and blood samples were withdrawn from the orbital sinus. Then, rats were killed through application of the cervical dislocation technique. After that, the liver, visceral adipose tissue, and epididymal adipose tissue were dissected. The liver was removed from each rat, washed with phosphate-buffered saline (ice cold, pH = 7.4) and weighed. Adipose tissue from each rat was also weighed. After standing for half an hour, blood samples were put in a centrifuge for 15 min at 2000 × *g* to obtain the sera which were stored in Eppendorf tubes at -80°C until the time of performing the laboratory analyses.

#### Measurement of Weight Gain, Liver Index and Adipose Tissue Index

Determination of the percent rise in body weight through the experiment (%BWt gain) was done using this formula: %BWt gain = (final BWt-Original BWt)/original BWt. Using the following equation [liver weight (g)/bodyweight (g)] × 100, the liver index was calculated. Furthermore, the adipose tissue index (ATI) was estimated using the following equation: ATI = [adipose tissue weight (g)/body weight (g)] × 100.

#### Measuring Serum Insulin, Leptin, Resistin, Adiponectin, and Inflammatory Cytokines by ELISA Kits

At end of the experiment, the serum level of insulin was estimated by a rat insulin ultrasensitive enzyme-linked immunosorbent assay (ELISA) kit (SunRed Bio, Shanghai, China). Serum level of leptin, resistin, adiponectin, TNF-α, and IL-1β were determined by rat ELISA kits from Cusabio Technology LLC (8400 Baltimore Avenue, MD, United States). Serum IL-6 was measured by an ELISA kit for rat IL-6 (RayBiotech Inc., Norcross, GA, United States). ELISA kits were used according to the instructions of the manufacturer.

#### Measuring Adipose Tissue Content of UCP-1 and CPT-1 by ELISA Kits

Frozen samples from the brown adipose tissue were used to prepare 10% tissue homogenate using cold phosphate-buffered saline (pH = 7.4). These homogenates were used to determine rat UCP-1 and CPT-1 using double-antibody sandwich ELISA kits from SunRed Biotechnology Company (Cat #. 201-11-1211 and 201-11-3700, Shanghai, China) according to the manufacturer’s protocol.

#### Estimation of Insulin Resistance and Insulin Sensitivity Indices

Insulin resistance was calculated by the homeostasis model assessment of insulin resistance (HOMA-IR). The applied equation was: HOMA-IR index = [FBG (mmol/L) × fasting insulin (μU/mL)]/22.5 as reported by Matthews et al. (1985). Insulin sensitivity was estimated by the revised-quantitative insulin sensitivity check index (R-QUICKI) equation: R-QUICKI = 1/[log fasting insulin (IU/mL) + log fasting glucose (mmol/L)] as described by Katz et al. (2000).

#### Measuring of Liver Function and Serum Lipid Profile

Commercial colorimetric kits purchased from Biocon Diagnostic (Germany) were employed to measure serum levels of alanine transaminase (ALT) and aspartate transaminase (AST). Serum total cholesterol (TC), low-density lipoprotein (LDL), high-density lipoprotein (HDL), and triglycerides (TGs) were measured by colorimetric kits from Biodiagnostic (Cairo, Egypt) using a Shimadzu spectrophotometer (UV-1601PC, Japan).

#### Quantitative Reverse Transcription–Polymerase Chain Reaction

Quantitative real-time PCR was employed for the determination of leptin, adiponectin, and visfatin expression in samples from the white adipose tissue and for assessment of UCP-1 and CPT-1 expression in brown adipose tissue. Extraction of the total cellular RNA from both white and brown adipose tissues was done using SV total RNA isolation system from Promega (Madison, United States), following the instructions of the manufacturer. The purity and concentration of extracted RNA were assessed using a Thermo Fisher Nanodrop NA-1000 UV/Vis spectrophotometer (Wilmington, DE, United States), and the extracted RNA was stored at -80°C. ΔC_*T*_ was determined against the expression of GAPDH gene as an internal control. Forward and reverse primers and annealing temperatures utilized for the PCR reactions are shown in **Table 1**. Quantitative real-time PCR was performed, while results were collected using GoTaq^®^ 1-Step RT-qPCR System from Promega (Madison, WI, United States) and the StepOnePlus^TM^ Applied Biosystems real-time PCR thermal cycler (MA, United States). The reaction mixture included 4 μL of RNA template, 1 μL of each of the two primers, 0.4 μL of GoScript^TM^ RT mix for 1-step RT-qPCR, 10 μL of GoTaq^®^ qPCR master mix, 0.31 μL of supplemental CXR reference dye, and 3.29 μL of nuclease-free water. The program consisted of reverse transcription at 37°C for 15 min and 10 min at 95°C to inactivate reverse transcriptase, then 40 cycles of denaturation at 95°C for 10 s, annealing for 30 s and finally extension for 30 s at 72°C.

**Table 1:** Primers and annealing temperatures used in real-time PCR reactions.

Gene	Primers	Annealing temperature
Leptin	Forward: 5′-GACATTTCACACACGCAGTC-3′	57°C
	Reverse: 5′-GAGGAGGTCTCGCAGGTT-3′	
Adiponectin	Forward: 5′-AATCCTGCCCAGTCATGAAG-3′	56°C
	Reverse: 5′-CATCTCCTGGGTCACCCTTA-3′	
Visfatin	Forward: 5′-CCTTACCTTAGAGTCATTCA -3′	52°C
	Reverse: 5′-GACATTCTCAATACTCCAC-3′	
UCP-1	Forward: 5′-CGATGTCCATGTACCACAAGGAA-3′	62°C
	Reverse: 5′-TCGCAGAAAAGAAGCCACAA-3′	
CPT-1	Forward: 5′-GGATGGCATGTGGGTAAAAG-3′	55°C
	Reverse: 5′-TACTGACACAGGCAGCCAAA-3′	
GAPDH	Forward: 5′-ATGACTCTACCCACGGCAAG-3′	56°C
	Reverse: 5′-GATCTCGCTCCTGGAAGATG-3′	

#### Microscopic Examination of Hematoxylin-Eosin Stained Adipose Tissue

Briefly, lumbar fat specimens were fixed in formalin-alcohol for 48 h. Then, fats were embedded in paraffin wax to prepare paraffin blocks and sliced into 3-mm sections for staining with hematoxylin and eosin (H&E). The stained fats were inspected by a light microscope (CX21; Olympus, Tokyo, Japan) and imaged at × 20. The diameter of 100 cells from two different microscopic fields was measured and used for calculation.

### Statistical Analysis

Data showed normal distribution were demonstrated as mean ± SE and tested for statistical differences using one-way analysis of variance (ANOVA) and Bonferroni’s test for multiple comparisons. Data related to measuring adipocyte diameters did not show normal distribution and therefore were presented in box-plots and compared by non-parametric ANOVA and Dunn’s test to compare every group with others. All possible comparisons were made among the experimental groups. *P*-values < 0.05 was considered significant. Version 17 of SPSS program (Chicago, IL, United States) and a trial version of GraphPad Prism were used to perform the statistical analysis.

## Results

Male rats fed with the HF diet for 16 weeks showed significant increases in %BWt gain versus normal rats fed on the normal diet (195 ± 15.6% vs. 55.3 ± 5.06%, **Table 2**). Treatment with RB (25 or 50 mg/kg) diminished the %BWt gain versus the HF diet control group. Further, RB (50 mg/kg) was more efficient in reducing the %BWt gain than the lower dose.

**Table 2:** Effect of russelioside B (25 and 50 mg/kg) on adiposity index, liver index and weight gain.

Groups	Adipose tissue index %	Liver index %	%BWt gain
Normal	2.19 ± 0.14	2.6 ± 0.14	55.3 ± 5.06
HF diet control	5.5 ± 0.49^∗^	3.47 ± 0.13^∗^	195 ± 15.6^∗^
HF diet + Russelioside B (25 mg/kg)	3.86 ± 0.38^∗#^	3.63 ± 0.35^∗^	126.7 ± 6.6^∗#^
HF diet + Russelioside B (50 mg/kg)	2.87 ± 0.23^∗#^	2.92 ± 0.14^#$^	108.9 ± 8.3^∗#$^

*BWt: body weight; HF: high fat. Percent weight gain was calculated as [(final BWt-initial BWt)/initial BWt] × 100. Results are mean ± SE and analyzed using one-way ANOVA and Bonferroni’s test at *P* < 0.05. ^∗^significant versus normal group, ^#^significant versus HF diet control, and ^$^significant versus HF diet + Russelioside B (25 mg/kg) group*.

Similar results were obtained from the adipose tissue and liver indices; the HF diet control rats showed nearly twice the value of ATI (5.5 ± 0.49 vs. 2.19 ± 0.14) and greater liver index (3.47 ± 0.13 vs. 2.6 ± 0.14) versus the normal group (**Table 2**). Treatment with RB (25 mg/kg) significantly reduced the ATI but not the liver index. However, the high dose of RB (50 m/kg) reduced both of the indices versus the HF diet control group (**Table 2**).

Measuring FBG and insulin levels demonstrated significant elevations in HF diet control group versus the normal group (98.23 ± 1.22 vs. 75.22 ± 1.54 and 45.12 ± 3.12 vs. 11.23 ± 1.12, respectively, **Table 3**). Similarly, the HOMA-IR index was greater and the QUICK index was lesser in the HF diet control group versus the normal group. Treatment with RB produced dose-dependent corrections in hyperglycemia and hyperinsulinemia as well as the high HOMA-IR index versus the HF diet control group. A similar correction was detected in QUICK index after treatment with RB (50 mg/kg). Importantly, results of the HF diet + RB (50 mg/kg) group were not significantly different from results of the normal group (**Table 3**).

**Table 3:** Effect of russelioside B (25 or 50 mg/kg) on fasting blood glucose, insulin, and insulin resistance indices.

Groups	FBG (mg/dL)	Insulin (μIU/L)	HOMA-IR	QUICKI (X 10)
Normal	75.22 ± 1.54	11.23 ± 1.12	2.11 ± 0.23	3.40 ± 0.32
HF diet control	98.23 ± 1.22^∗^	45.12 ± 3.12^∗^	11.12 ± 1.23^∗^	2.77 ± 0.30^∗^
HF diet + Russelioside B (25 mg/kg)	85.34 ± 0.99^∗#^	32.54 ± 2.56^∗#^	7.23 ± 1.32^∗#^	2.90 ± 0.31^∗^
HF diet + Russelioside B (50 mg/kg)	78.12 ± 1.45^#$^	17.22 ± 2.23^#$^	3.12 ± 1.12^#$^	3.22 ± 0.40^∗#$^

*FBG, fasting blood glucose; HF, high fat. Data are represented as mean ± SE. Comparisons were performed by one-way ANOVA and Bonferroni’s test for multiple comparisons at *P* < 0.05. ^∗^significant versus normal group, ^#^significant versus HF diet control, and ^$^significant versus HF diet + Russelioside B (25 mg/kg) group*.

Measuring serum lipids highlighted greater TC, TGs, and LDL in HF diet control rats versus normal rats however, lower HDL-C value was found. The two employed doses of RB produced a dose-dependent decrease in the elevated serum TGs level. Differently, serum TC, LDL, and HDL-C were modulated significantly only by treatment with the higher dose of RB (**Table 4**). Serum ALT and AST activities were higher in HF diet control rats versus normal rats. Treatment with both doses of RB successfully reduced the elevated liver enzyme activities; the effect on both enzymes was dose-dependent (**Table 4**).

**Table 4:** Effect of russelioside B (25 and 50 mg/kg) on serum lipid profile and liver enzyme activities in rats fed with high fat diet.

Groups	TGs mg/dL	TC mg/dL	LDL mg/dL	HDL mg/dL	ALT IU/L	AST IU/L
Normal	150.22 ± 2.88	90.34 ± 2.11	55.12 ± 1.23	41.22 ± 1.87	50.12 ± 2.33	69.12 ± 5.23
HF diet control	222.34 ± 3.66^∗^	120.33 ± 3.22^∗^	69.24 ± 2.12^∗^	30.12 ± 1.65^∗^	78.23 ± 3.23^∗^	90.14 ± 6.87^∗^
HF diet + Russelioside B (25 mg/kg)	182.33 ± 3.13^∗#^	111.34 ± 2.32^∗^	63.23 ± 1.44^∗^	33.34 ± 1.34^∗^	65.12 ± 4.23^∗#^	81.65 ± 5.18^∗#^
HF diet + Russelioside B (50 mg/kg)	160.23 ± 3.45^#$^	94.31 ± 2.23^#$^	58.12 ± 2.14^#^	38.12 ± 1.87^#$^	54.33 ± 5.12^∗#$^	72.23 ± 5.77^#$^

*ALT, alanine transaminase; AST, aspartate transaminase; HDL, high-density lipoprotein; LDL, low-density lipoprotein; TC, total cholesterol; TGs, triglycerides. Data are represented as mean ± SE. Comparisons were performed by one-way ANOVA and Bonferroni’s test at *P* < 0.05. ^∗^ significant versus normal group, ^#^ significant versus HF diet control, and ^$^significant versus HF diet + Russelioside B (25 mg/kg) group*.

**FIGURE 2:**
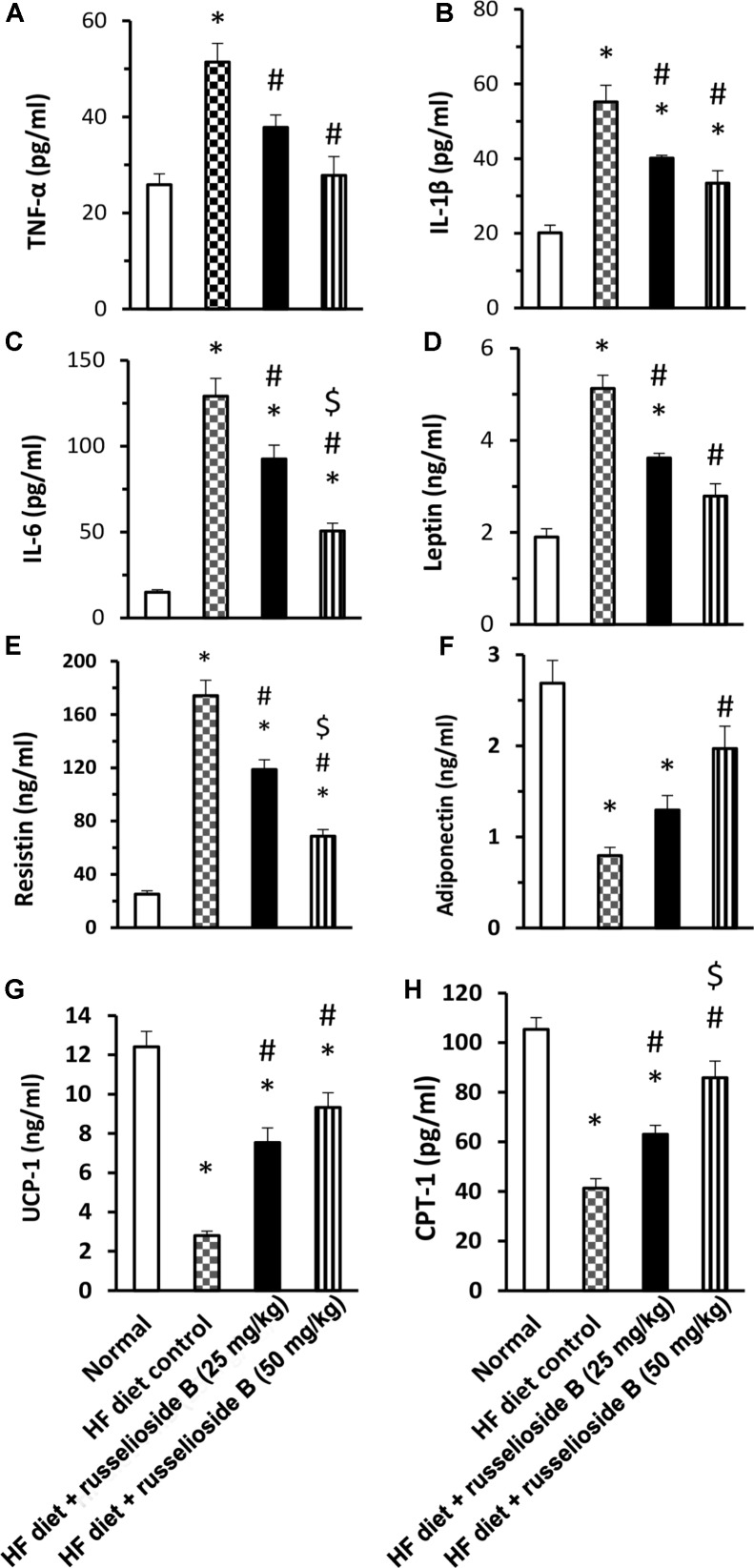
Effect of russelioside B on serum level of adipokines and inflammatory cytokines in high fat diet fed rats. **(A)** TNF-α; **(B)** IL-1β; **(C)** IL-6; **(D)** leptin; **(E)** resistin, **(F)** adiponectin, **(G)** uncoupling protein-1, and **(H)** carnitine palmitoyl transferase-1 in the study groups. Data are expressed as mean ± SE. ^∗^significant versus normal group, ^#^significant versus HF diet control group, and ^$^significant versus HF diet + russelioside B (25 mg/kg) group. Comparison was set by one-way ANOVA and Bonferroni’s test at *P* < 0.05.

The results of serum ELISA assays are demonstrated in **Figure 2**. The results indicated higher levels of TNF-α, IL-6, and IL-1β in the HF diet control group versus the normal group (**Figures 2A–C**). Treatment with RB (25 and 50 mg/kg) significantly decreased these markers versus the HF diet control group. Further, HF diet control rats showed higher serum leptin and resistin, but lower adiponectin level as compared to the normal group. RB (25 or 50 mg/kg) significantly reduced serum leptin and resistin versus the HF diet control group (**Figures 2D,E**). The high dose of RB (50 mg/kg) successfully improved serum adiponectin versus the HF diet control group (**Figure 2F**). The measured proteins for UCP-1 and CPT-1 in adipose tissue indicated significant declines in the HF diet control group. Oral treatment with RB at both doses increased the level of UCP-1 and CPT-1 versus the HF diet control (**Figures 2G,H**). Interestingly, the high dose of RB increased CPT-1 level versus the low dose of RB.

**FIGURE 3:**
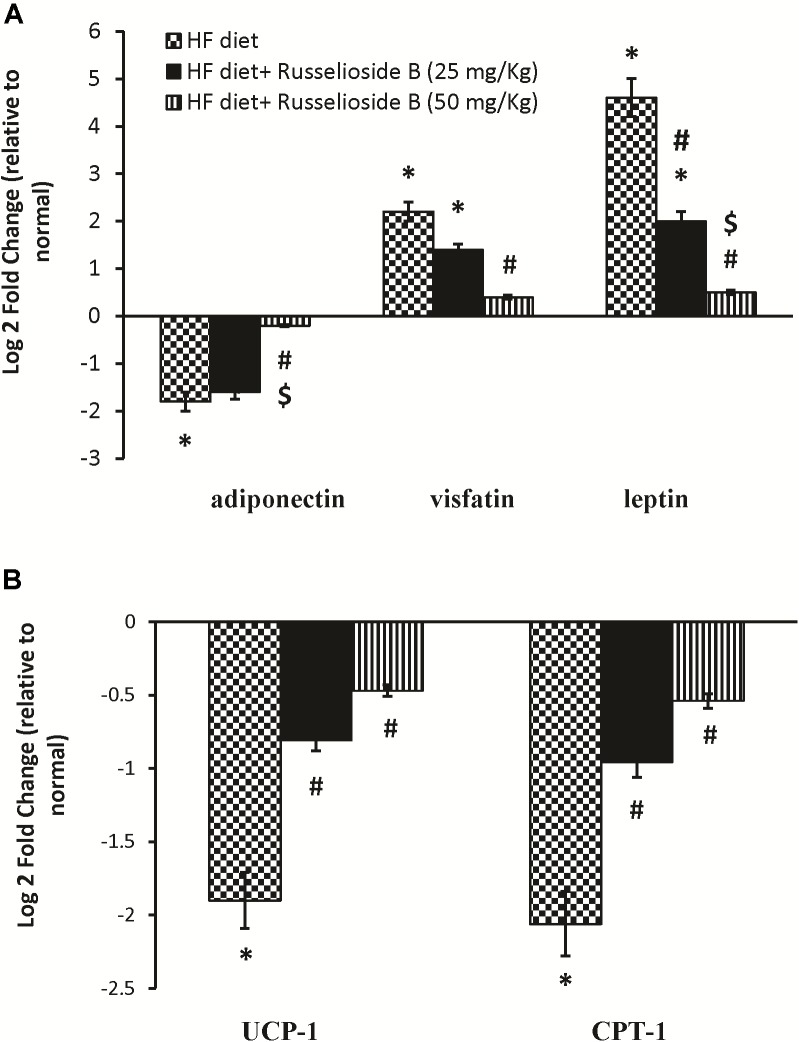
Effect of russelioside B on the mRNA expression of adipokines and energy expenditure genes in adipose tissue using quantitative real-time PCR. **(A)** Gene expression of adiponectin, visfatin, and leptin in white adipose tissue; **(B)** gene expression of mitochondrial uncoupling protein-1 (UCP-1) and carnitine palmitoyl transferase 1 (CPT-1) in brown adipose tissue. Data are expressed as log2 fold change relative to the normal group. Negative values indicate downregulation. Bars correspond to standard error. ^∗^significant versus normal group, ^#^significant versus HF diet control group, and ^$^significant versus HF diet + russelioside B (25 mg/kg) group. Comparison was done by one-way ANOVA followed by Bonferroni’s test at *P* < 0.05.

**Figure 3A** shows the mRNA expression of leptin, adiponectin, and visfatin in samples from the white adipose tissue. Adiponectin expression was downregulated in HF diet control rats versus normal rats (30% decrease). Treatment with the high dose of RB significantly upregulated the mRNA expression of adiponectin compared to the HF diet controls (**Figure 3A**). On the other hand, mRNA expression of leptin and visfatin was upregulated in the HF diet control rats versus the normal rats. Both doses of RB effectively downregulated leptin and visfatin expression versus the HF diet control group (**Figure 3A**). Additionally, UCP-1 and CPT-1 genes were downregulated in the brown adipose tissue of HF diet control rats. Treatment with RB upregulated both genes significantly (**Figure 3B**).

**FIGURE 4:**
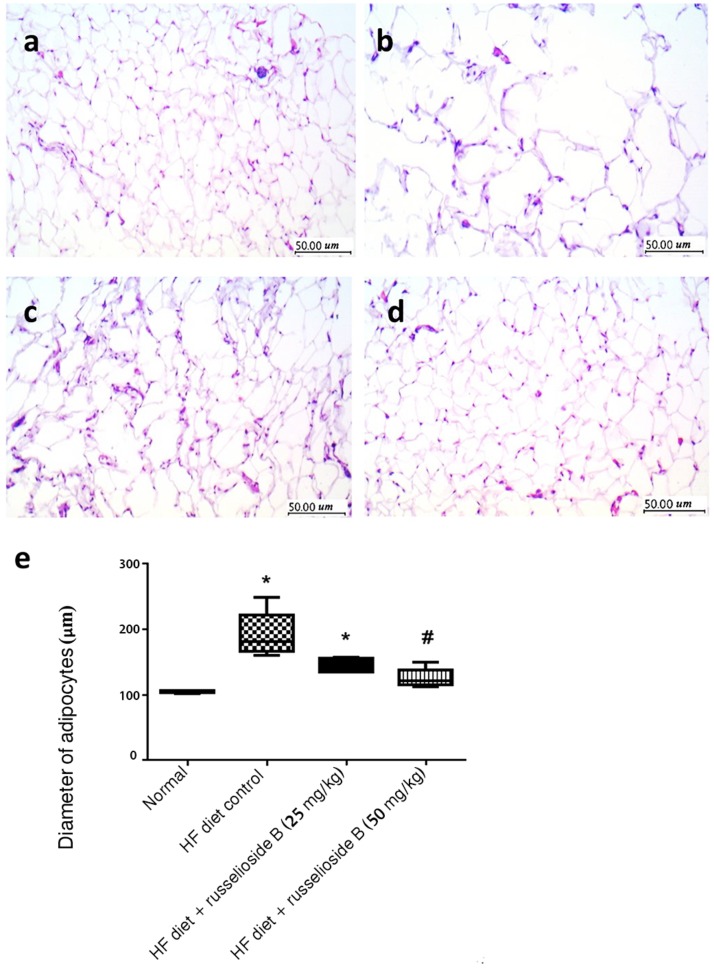
Effect of russelioside B on the diameter of adipocyte spaces in the lumbar adipose tissue. **(a)** Tissue section from normal rat adipose tissue showing low-diameter adipocyte space; **(b)** tissue section from adipose tissue from HF diet control rats showing larger-diameter adipocyte spaces; **(c, d)**: tissue sections from adipose tissues from HF diet fed rats treated with RB at 25 and 50 mg/kg, respectively; and **(e)**: box plots showing the medians and quartiles for diameters (μm) of adipocyte spaces. ^∗^significant versus normal group, ^#^significant versus HF diet control group, and ^$^significant versus HF diet + russelioside B (25 mg/kg) group. Comparison was performed by non-parametric ANOVA followed by Dunn’s test at *P* < 0.05.

**Figures 4a–d** showed tissue sections from the adipose tissues. The adipose tissue from HF diet control rats showed larger-diameter for adipocyte spaces. Treatment with RB (50 mg/kg) significantly reduced the diameter of the adipocyte spaces (**Figure 4e**).

## Discussion

Obesity is an intricate medical problem challenges both medical professionals and health bodies. Natural medicine if precisely explored represents a potential supportive and/or alternative strategy for long-term therapies of obesity (Abdel-Sattar et al., 2014). *C. quadrangula* has been used traditionally as antidiabetic and appetite-suppressant (Gushash, 2006). RB (a pregnane glycoside) was first isolated from *C. russeliana* in a considerable yield; >3.4% of the dry powder (Al-Yahya et al., 2000). Besides, RB is further isolated and quantified by LC-MS (4.8% of dry powder) in *C. tuberculata* and *C. quadrangula* (Abdel-Sattar et al., 2013, 2016).

In the current model of HF feeding in rats for 16 weeks, we observed (i) increased % BWt gain and ATI, (ii) elevated liver enzymes, and (iii) increased systemic insulin resistance. These data are in line with data produced previously (Zaitone and Essawy, 2012; Tunapong et al., 2018). In agreement, human studies highlighted a causal relationship between excessive caloric intake, weight gain, and tissue resistance to insulin (Delgado et al., 2018). Furthermore, a plethora of data reports the interplay of pro-inflammatory cytokines and adipokines in the pathology of insulin resistance and associating comorbidities (Hotamisligil et al., 1993; Gregor and Hotamisligil, 2011; Alcalá et al., 2017). These findings are in line with the data presented herein, where circulating IL-1β, IL-6, and TNF-α significantly increased in obese animals.

Adipokines play a crucial rule in homeostasis of the adipose milieu (Rabe et al., 2008). The current results indicated that HF diet fed rats exhibited decreased levels of circulating adiponectin and downregulation of its gene expression. Oppositely, hyperleptinemia was detected with upregulation of its gene expression in agreement with some data presented previously (Arita et al., 1999; Trayhurn and Beattie, 2001; Almabrouk et al., 2018). Interestingly, adiponectin level is inversely but leptin is directly correlated to obesity and insulin resistance (Koleva et al., 2013). In excess caloric states, leptin traffics to the hypothalamus to suppress appetite and improve thermogenesis (Alcalá et al., 2017). Similar to leptin, the current results highlighted greater serum resistin level in HF diet control rats. Resistin is secreted by mature adipocytes, controls the systemic insulin sensitivity (Steppan et al., 2001). One study revealed that recombinant resistin hinders glucose tolerance and insulin actions in regular mice. Other studies revealed that resistin was found in positive correlation with the HOMA-IR index in obese subjects (Silha et al., 2003) and in DIO model (Steppan et al., 2001). In contrast, administration of anti-resistin antibody recovers blood glucose and insulin effects in mice with DIO (Steppan et al., 2001). In the current study, resistin level was favorably modulated by RB. Similarly, resistin expression was reported to be suppressed in response to the insulin sensitizer, rosiglitazone (Lehmann et al., 1995; Steppan et al., 2001); this was explained by suppressing expression of resistin.

Visfatin is a pro-inflammatory and insulin-mimetic mediator. In the current study, visfatin mRNA expression significantly increased in white adipose tissue of obese rats. In support, a clinical study demonstrated a direct correlation between plasma visfatin, body mass index, and visceral adiposity (Berndt et al., 2005). In contrary, other researchers demonstrated that visfatin is directly correlated to inflammatory states (Auguet et al., 2013). Importantly, RB ameliorated the perturbed metaflammatory profile by reducing TNF-α, IL-6, IL-1β, leptin, resistin, and visfatin with increasing adiponectin. Measuring mRNA expression of leptin and adiponectin are consistent with their serum levels.

This study aim was to determine the potential therapeutic benefits of RB in diminishing weight gain in HF diet-fed rats. Interestingly, our data showed that RB (25 and 50 mg/kg) improved lipid profile, reduced weight gain and insulin resistance as indicated by the HOMA-IR index. Similarly, experimental studies reported that RB at the same doses used in this study alleviated hyperglycemia and hyperlipidemia in the streptozotocin model in rats. Authors highlighted that RB regulated enzymes involved in carbohydrate metabolism in the liver of diabetic rats (Abdel-Sattar et al., 2016).

Furthermore, our data revealed upregulation of both UCP-1 and CPT-1 genes and increased their protein level in the brown adipose tissue in rats treated with RB. CPT-1 is the rate-limiting enzyme in mitochondrial long-chain fatty acids β-oxidation, which is crucial for maintaining of the brown adipose tissue (Gonzalez-Hurtado et al., 2018). In rodent models, activation of brown adipose tissue was suggested to improve glucose homeostasis and ameliorate insulin resistance (Peirce and Vidal-Puig, 2013; Zaitone et al., 2015). CPT-1 upregulation is well-known to play a role in obesity alleviation (Rameshreddy et al., 2018). Upregulation of both genes may be also linked to enhanced lipolysis and decreased TGs level, where the released free fatty acids are transported to the mitochondria via the activated CPT-1 enzyme, with simultaneous induction of the thermogenic activity of UCP-1 (Bargut et al., 2016). UCP-1 is known to mediate diet-induced adrenergic thermogenesis, where its ablation was found to induce obesity (Feldmann et al., 2009). Moreover, activation of UCP-1 altogether with increased lipolysis was suggested to reduce visceral fat mass (Han et al., 2017).

## Conclusion

Russelioside B isolated from *C. quadrangula* was studied for its beneficial therapeutic role in a rat model of dietary obesity and insulin resistance. RB controlled weight gain, improved lipid profile, and ameliorated inflammatory derangement accompanying DIO and insulin resistance. Further, RB modulated adipokine expression and increased expression and protein level of energy expenditure enzymes. Therefore, the overall antiobesity action of RB may be, at least partly, attributed to its anti-inflammatory and adipokine modulating activities in addition to it is favorable effect on energy expenditure. Future studies are warranted to investigate the pharmacological actions of RB on important organs such as the liver and to fully explore its compensatory mechanisms against the metabolic effects of HF feeding in rats.

## Author Contributions

EA-S set the main idea of the manuscript, collected the plant material, isolated and structurally identified the RB, and participated in the design of the experiment and writing and submitting the manuscript. EM participated in the design of the experiment, followed the experimental animals and performed the biochemical assays, interpreted the results of biomarkers, and participated in writing and discussion of the result. SE-G participated in the design of the experiment, discussion of the result, and writing of the manuscript. HM participated in the design of the experiment, followed the experimental animals, discussed the result, and participated in writing. HE participated in the design of the experiment, histopathological examination, discussion of the result, and writing of the manuscript. SZ participated in the design of the experiment, followed the experimental animals, interpreted the results of biomarkers, discussion of the result, and participated in writing.

## Conflict of Interest Statement

The authors declare that the research was conducted in the absence of any commercial or financial relationships that could be construed as a potential conflict of interest. The reviewer BS-C and handling Editor declared their shared affiliation at the time of the review.

## Supplementary Material

The Supplementary Material for this article can be found online at: https://www.frontiersin.org/articles/10.3389/fphar.2018.00990/full#supplementary-material

FIGURE S1 ^1^H-NMR spectrum of russelioside B.

FIGURE S2 ^13^C-NMR spectrum of russelioside B.
