# Supplementary figures and images for: Pharmacological Action of a Pregnane Glycoside, Russelioside B, in Dietary Obese Rats: Impact on Weight Gain and Energy Expenditure
Source: Front Pharmacol. 2018 Aug 30;9:990. doi: 10.3389/fphar.2018.00990 (PMC6125411; doi:10.3389/fphar.2018.00990)

Dr-Essam AbdElSatar\_H\_1  
Dr-Essam AbdElSatar\_H\_1

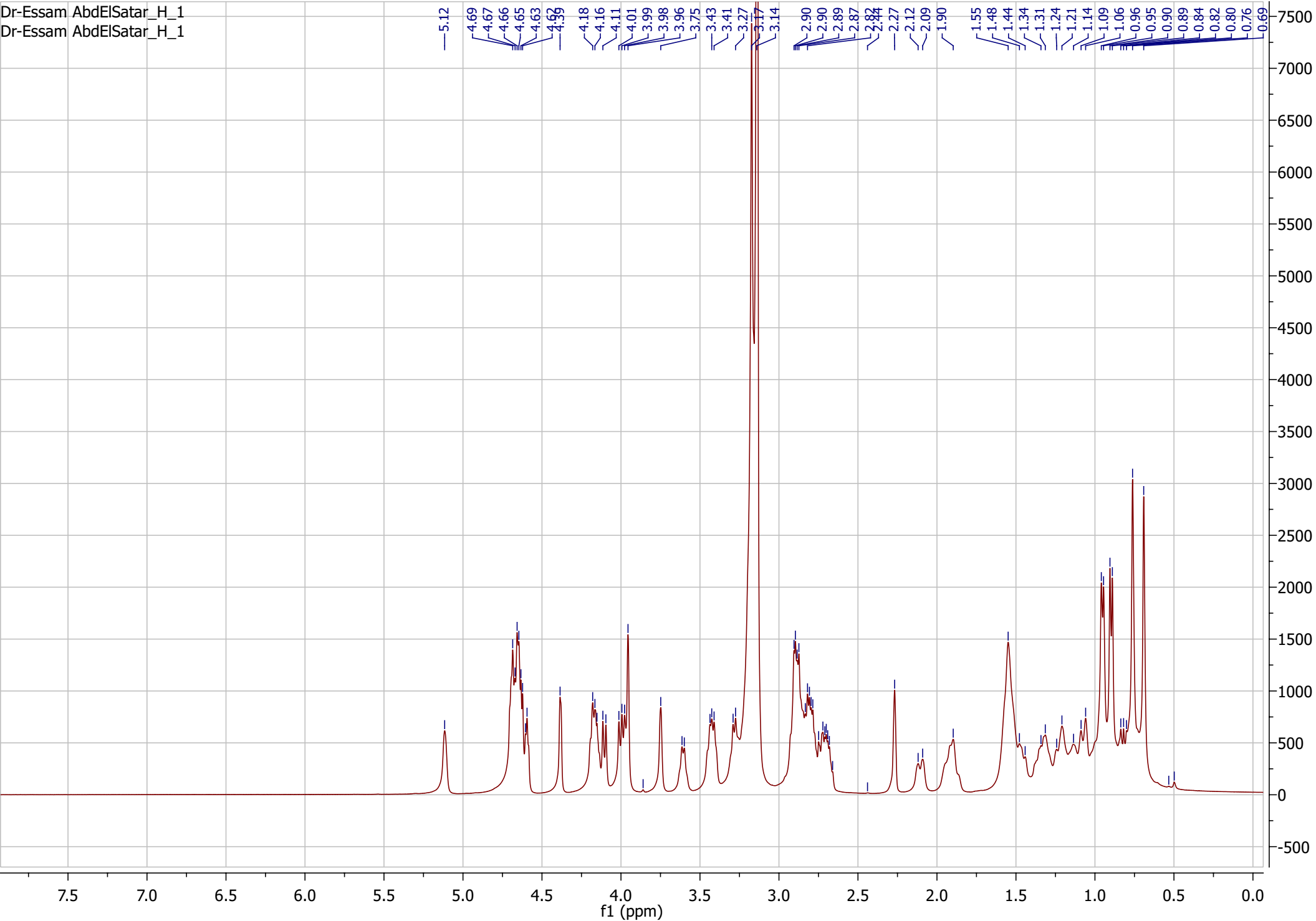

Supplement: FIGURE S1 — 1H-NMR spectrum of russelioside B. [file Image_1.PDF]

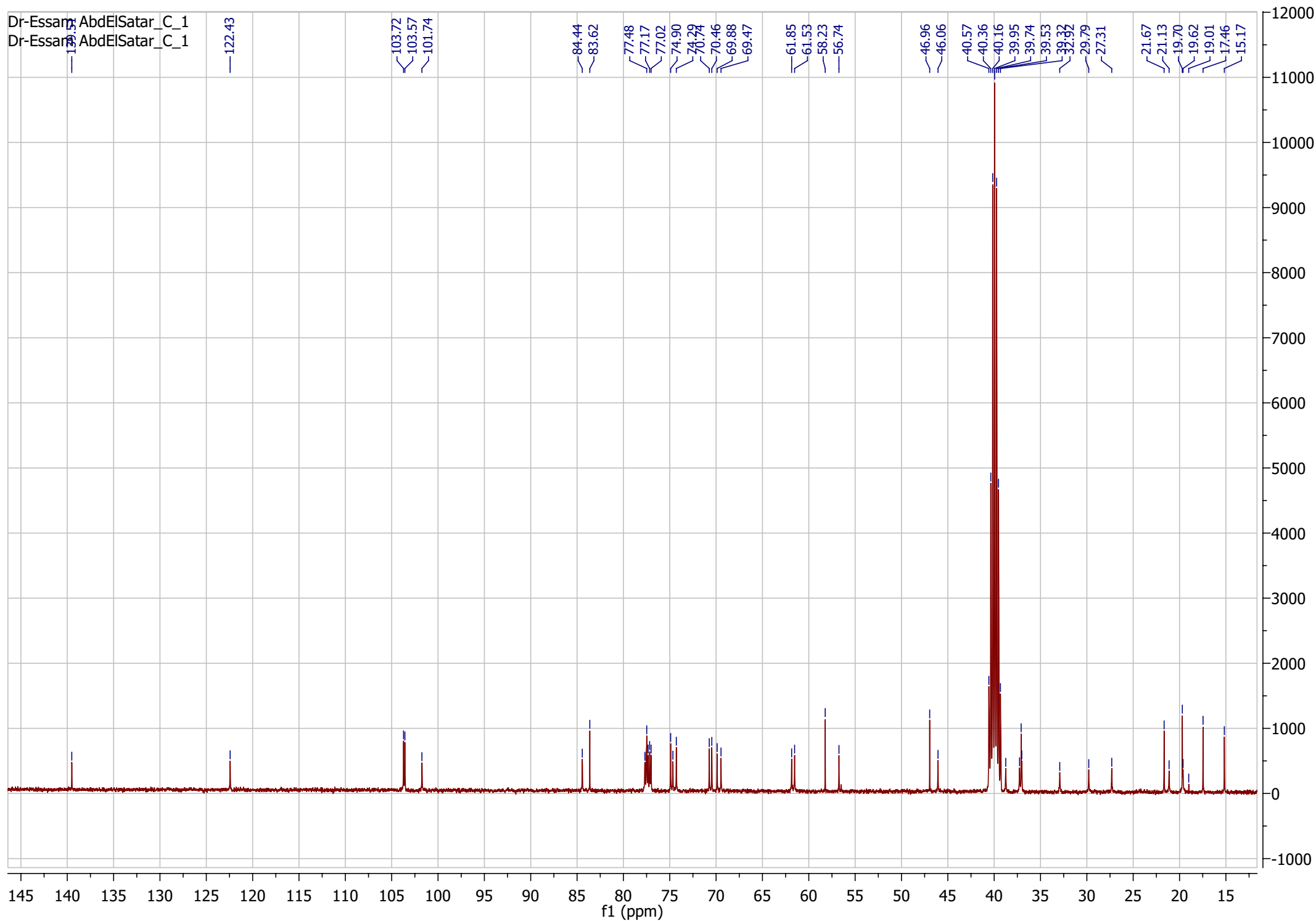

Supplement: FIGURE S2 — 13C-NMR spectrum of russelioside B. [file Image_2.PDF]
